# Diagnostic and Management Pathways for Pulmonary Carcinoid Tumours in the United Kingdom: Results from the National Lung Neuroendocrine Tumour Pathway Project

**DOI:** 10.1155/2020/9287536

**Published:** 2020-02-28

**Authors:** Wasat Mansoor, Stuart Ferguson, Victoria Ross, Denis Talbot

**Affiliations:** ^1^The Christie NHS Foundation Trust, Wilmslow Rd., Manchester M20 4BX, UK; ^2^Novartis Pharmaceuticals UK Limited, 2nd Floor, The WestWorks Building, White City Place, 195 Wood Lane, London W12 7FQ, UK; ^3^Department of Oncology, University of Oxford, Oxford OX3 7DQ, UK

## Abstract

There is inconsistency among published guidelines for the optimal diagnostic and management pathways for patients with typical (TC) or atypical (AC) pulmonary carcinoid tumours. We conducted a UK-wide clinician survey to assess current practice for the diagnosis, management, and follow-up of patients with TC/AC and descriptively compared management between European Neuroendocrine Tumor Society (ENETS) accredited centres of excellence (CoE) and nonaccredited centres (non-CoE). Twenty-seven clinicians (10 CoE; 17 non-CoE) participated. Computed tomography of thorax, abdomen, and pelvis was the most commonly reported diagnostic tool (96% of respondents), and bone scans and gallium somatostatin receptor scintigraphy positron emission tomography (SRS PET) were the least commonly reported (30% and 37% of respondents, respectively). Adjuvant therapy is considered for resected TC/AC by <5% of respondents for patients with stage N0 M0 AC or TC, up to 48% of respondents for patients with AC with R1 disease. Somatostatin analogues were the most commonly reported first-line treatment (63% of respondents), and chemotherapy was the most commonly reported second-line therapy and third-line therapy (33% and 41%, respectively) for unresectable and metastatic disease. Reported frequency of initial follow-up after primary surgery ranged from every 2 months to annual, and total follow-up duration ranged from 2 years to indefinite depending on disease type (TC/AC) and stage. For most diagnostic investigations, the highest reported frequency of use was in CoE, most notably gallium SRS PET (70% CoE vs. 18% non-CoE respondents). 93% of respondents (100% CoE; 88% non-CoE) reported having neuroendocrine tumour- (NET-) specialist multidisciplinary team meetings at their centre; 59% (90% CoE; 41% non-CoE) had a NET Clinical Nurse Specialist (CNS) and 48% (80% CoE; 29% non-CoE) had a lung NET patient database. The survey results suggest variability between UK centres in diagnostic pathways and management of patients with TC/AC and suggest that CoE may be able to offer an improved service to patients.

## 1. Introduction

Pulmonary carcinoid tumours (PC), a subset of pulmonary neuroendocrine tumours (NETs), are rare neuroendocrine epithelial malignancies [1, 2]. PC are classified as well-differentiated low-grade (or grade 1) typical carcinoid tumours (TC) and poorly differentiated intermediate-grade (or grade 2) atypical carcinoid tumours (AC) [1–5]. Despite having a high overall survival rate, TCs metastasise in 10–15% of cases and ACs in up to 50% [6, 7], with the potential for recurrence to occur many years after completion of primary treatment [8].

Accurate and timely diagnosis of PC can determine the success of treatment as each subtype requires a specific treatment approach [2, 9]. This can, however, be challenging as patients are often asymptomatic or show nonspecific symptoms at presentation [9, 10]. In addition, TC and AC have subtle histopathological differences [11, 12] that make accurate diagnosis more challenging. These factors may lead to delay in diagnosis or initial misdiagnosis [13, 14]. Patients with AC and TC require different treatment approaches [15], emphasising the importance of employing a clear diagnostic strategy involving the use of appropriate diagnostic tools [16].

There are inconsistencies in available guidelines for the diagnostic and clinical management pathways for patients with PC [8, 17–19], including recommendations for radiation and systemic treatment resulting in a lack of consensus on the use of adjuvant treatment after complete surgical resection [8] and on the standard of care for unresectable or metastatic tumours [9, 11]. Some available guidelines are not specific to, or do not distinguish between, patients with TC and AC [17], and some offer advice related to the predominant forms of pulmonary NETs (i.e., small-cell lung cancer) and not to TC and AC [19]. Another challenge is that, in the absence of well-designed, randomised controlled clinical trials on TC and AC, several nonvalidated treatment approaches have evolved in single centres [20, 21], and most available literature and guidelines are based on lower-level evidence, case studies, or mixed populations of patients with pulmonary NETs [8]. It is thought that these challenges may lead to variability in the delivery of patient care; this is supported by research conducted on patients with pulmonary NETs about their experiences of diagnosis and treatment, in which patients stated having “no clear care pathway” and reported difficulties with obtaining a diagnosis, finding appropriate information about NETs from healthcare professionals, and accessing disease-specific support [22].

In an effort to standardise and improve the management of NETs, ENETS initiated a certification process for NET “centres of excellence” (CoE) in 2008, focusing primarily on gastroenteropancreatic (GEP) NETS. The aim was to establish a network of accredited centres across Europe that are specialised in NET diagnosis and management [23]. To be eligible for CoE accreditation, centres must meet a minimum threshold for the number of patients diagnosed with GEP NETs at the centre per year and undergo a rigorous certification process including audits of service organisation, staffing, and structure. Benefits of ENETS CoE accreditation, reported by certified centres, include having improved patient documentation, multidisciplinary team (MDT) cooperation, better patient follow-up, and increased participation in research [23].

In order to establish consensus for the diagnosis and management of PC, ENETS published guidelines in 2015 [8] which emphasise the importance of accurate and timely diagnosis and a multidisciplinary approach to patient care. However, published data on the pathways to diagnosis and management of patients with TC and AC in routine clinical practice in order to evaluate the implementation of these guidelines is sparse.

In this context, we conducted a UK-wide survey of clinicians to assess current practice for the diagnosis, management, and follow-up of patients with TC and AC in UK and a narrative comparing patient management pathways between UK CoE and non-CoE with the overall aim of identifying areas for future service improvement.

## 2. Materials and Methods

A bespoke survey was designed and developed by the investigators based on perceived knowledge gaps in the current TC and AC management pathway. The survey questions were developed by their medical team and validated by a NET-specialist clinician. The survey consisted of 22 questions (combination of fixed-choice and open-ended [free-text] questions), which were based on topics including the epidemiology, diagnostics, and treatment pathways for lung NETs, focusing on TC and AC. The results of the survey were intended to describe centre-level management rather than the practices of the individual respondents.

It was planned to include approximately 30 UK-based clinicians in the survey (no more than one per centre), aimed at good geographic coverage and representation from all ten UK CoE to reduce the risk of bias. Ninety-five clinicians with responsibility for managing patients with TC and AC were initially identified by the sponsor project clinical leads (existing contacts and via hospital/NHS Trust websites) and invited by e-mail to participate in the survey. Clinicians were requested to forward the e-mail invitation to other more suitable colleagues if they felt they were not best placed to participate. The response rate and respondent profile were evaluated on an ongoing basis after the initial invitation e-mail, and a number of additional clinicians were subsequently invited to participate, specifically targeting geographic regions or CoE that were not yet represented. Clinicians were also recruited via an advertisement placed on the UK and Ireland Neuroendocrine Tumour Society (UKI NETS) members' website. All clinicians who provided written informed consent to participate were included in the survey.

Interviews with the participating clinicians, to gather responses to the survey questions, were conducted between October 2016 and May 2017 by telephone, either by researchers from pH Associates (now trading as OPEN VIE, an independent healthcare research company) or members of the Novartis Medical Science Liaison (MSL) team. In total, five people were involved in conducting the interviews, and training was provided before the project commenced. The first interview was conducted jointly with two researchers to facilitate consistency, with the remaining interviews conducted on a one-to-one basis. The investigators were informed that research ethics approval was not required for this clinician-based survey [24].

### 2.1. Data Analysis

Quantitative variables are reported using descriptive statistics of distribution, central tendency, and dispersion, as appropriate to the data collected. Categorical variables are described with frequency and percentages. Free-text (open-ended) questions were analysed by content analysis; this involved an initial review of the text to identify important concepts, followed by coding and categorisation into themes under the broad headings provided by the structure of the survey questions. When appropriate, results are shown separately for accredited CoE versus non-CoE. Not all respondents answered every question.

## 3. Results

Twenty-seven clinicians from 27 secondary and tertiary care centres in England (*n* = 22), Scotland (*n* = 3), Wales (*n* = 1), and Northern Ireland (*n* = 1) participated in this survey, with a geographically balanced spread of respondents across England. All of the ten UK ENETS CoE that were accredited at the time of undertaking the survey were represented. Of the 27 participating clinicians, 37% (10/27) were from CoE and 63% (17/27) were from non-CoE. The participating clinicians were oncologists (*n* = 4 at CoE; *n* = 15 at non-CoE), respiratory specialists (*n* = 2 at non-CoE), and other specialists (gastroenterologists, endocrinologists, hepatologists, and surgeons, *n* = 6 at CoE).

Of the 27 respondents, 18 provided estimates of the number of new patients presenting to their centres with TC or AC per year, with estimates ranging between 2 and 80 overall (6–80 patients per year for CoE respondents and 2–29 patients per year for non-CoE respondents). The estimated proportion of new patients presenting with advanced (unresectable or metastatic) disease (median) was 10% for TC and 30% for AC.

### 3.1. Diagnostic Pathways

**Figure 1:**
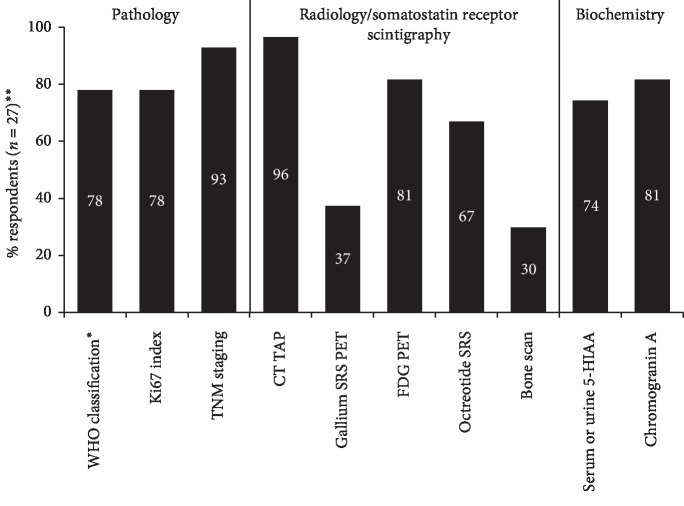
Reported use of tests and investigations at diagnosis. ^*∗*^Any version. ^*∗∗*^Bars represent the percentage of respondents reporting use of tests/investigations at their centre; the remainder reported either ‘no' (i.e., test not used) or ‘not known.' Abbreviations: 5-HIAA, 5-hydroxyindoleacetic acid; CT TAP, computed tomography of thorax, abdomen, and pelvis; FDG, fluorodeoxyglucose; PET, positron emission tomography; SRS, somatostatin receptor scintigraphy; TNM, tumour node metastasis; WHO, World Health Organisation.

The reported use of different tests and investigations at diagnosis across all respondents is shown in Figure 1. Overall, the most commonly reported (by >75% of the respondents) tests and investigative tools were computed tomography of thorax, abdomen, and pelvis (CT TAP, reported by 96% of the respondents), the Tumour, Node, Metastasis (TNM) staging system (93% of respondents), fluorodeoxyglucose (FDG) positron emission tomography (PET) (81% of respondents), chromogranin A (81% of respondents), the Ki67 index (78% of respondents), and the World Health Organisation (WHO) classification system (78% of respondents). Bone scans and gallium somatostatin receptor scintigraphy (SRS) PET were the least commonly used diagnostic procedures (30% and 37% of respondents, respectively). One-third of respondents (9/27, 33%) reported that both FDG PET and gallium SRS PET are used for diagnosis at their centre.

### 3.2. Treatment and Disease Management

The number and proportion of respondents who would consider offering various types of adjuvant treatment (including somatostatin analogues [SSA], chemotherapy, and radiotherapy) after surgery to patients with resected TC/AC by disease stage, across all centres, is shown in Table 1. Patients with TC or AC at N0 M0 stage were least likely to be considered for any form of adjuvant treatment (<5% of respondents). For patients with N1 M0 stage tumours, 33% of the respondents reported considering some form of adjuvant treatment for patients with AC and 15% for patients with TC. For patients with N2-3 M0 stage tumours, 48% of respondents reported considering some form of adjuvant treatment for patients with AC and 30% for patients with TC. For those patients with residual disease after resection (R1 resected), 48% of respondents reported considering any form of adjuvant treatment for patients with AC and 41% for patients with TC.

First, second- and third-line treatments for patients with advanced (unresectable or metastatic) disease across all centres are shown in Table 2. The most commonly reported first-line treatments were SSAs (63% of respondents), surgery (22%), and chemotherapy (11%); the most commonly reported second-line treatments were chemotherapy (33%), mTOR inhibitors/targeted therapy (26%), and SSAs (19%); and the most commonly reported third-line treatments were chemotherapy (41%), peptide receptor radionuclide therapy (PRRT) (19%), and mTOR inhibitors/targeted therapy such as everolimus (11%).

### 3.3. Variations in Follow-Up

Reported frequency of initial follow-up and total duration of follow-up for patients with PC after completion of their initial treatment varied according to type (TC or AC) and tumour stage (Table 3). For example, reported follow-up duration for patients with more advanced AC (at stage N2-3 M0 and above) ranged from 2 years to indefinite.

**Figure 2:**
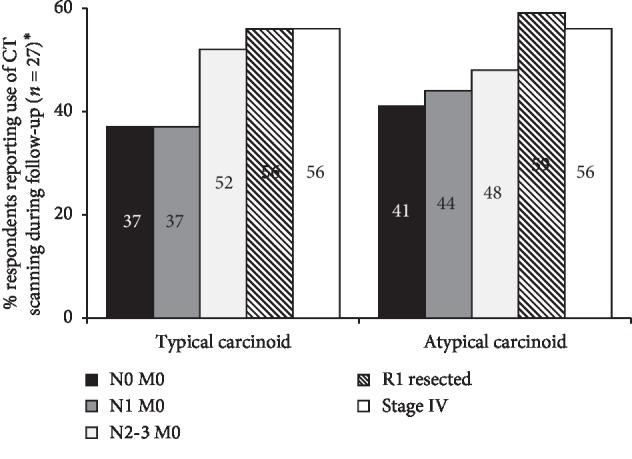
Reported use of CT scanning during follow-up. ^*∗*^Information about current follow-up schedules was ascertained using an open question asking the respondents how patients with TC/AC at their centre are followed up at different stages of disease. Bars represent the percentage of respondents who mentioned use of CT scanning as part of their response.

For both TC and AC, respondents most commonly reported the use of CT scanning during follow-up in patients with tumours at stage N2-3 M0 and above (Figure 2).

Overall, 30% (8/27) of respondents reported using a more intensive follow-up schedule (i.e., more frequent, longer duration or greater use of scans) for patients with AC compared to those with TC.

Sixty-seven percent (18/27) of respondents reported the possibility of patients being lost to follow-up (LTFU) under their current management pathways. Reasons cited for this included patients becoming lost to follow-up after surgery (for example, if they are discharged without being referred to an appropriate specialist team/MDT), patient nonattendance at follow-up appointments (due to the length of follow-up or age-related comorbidities), and the perception by clinicians of TC/AC as slow-growing/low-risk tumours, which may lead to a less stringent follow-up schedule.

### 3.4. Descriptive Comparison of Diagnosis and Management between CoE and Non-CoE

**Figure 3:**
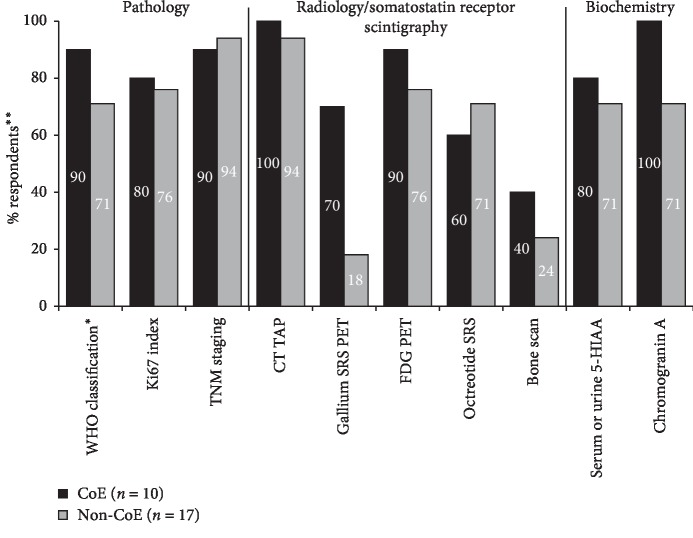
Reported use of tests and investigations at diagnosis in CoE vs. non-CoE respondents. ^*∗*^Any version. ^*∗∗*^Bars represent the percentage of respondents reporting use of tests / investigations at their centre; the remainder reported either ‘no' (i.e., test not used) or ‘not known.' Abbreviations: 5-HIAA, 5-hydroxyindoleacetic acid; CoE, Centre of Excellence; CT TAP, computed tomography of thorax, abdomen and pelvis; FDG, fluorodeoxyglucose; PET, positron emission tomography; SRS, somatostatin receptor scintigraphy; TNM, Tumour Node Metastasis; WHO, World Health Organisation.

Reported use of different tests and investigations at diagnosis in CoE and non-CoE is shown in Figure 3. For the majority of tests, the highest reported frequency of use was in CoE, most notably gallium SRS PET, which was reported by 70% of CoE and 18% of non-CoE respondents.

**Figure 4:**
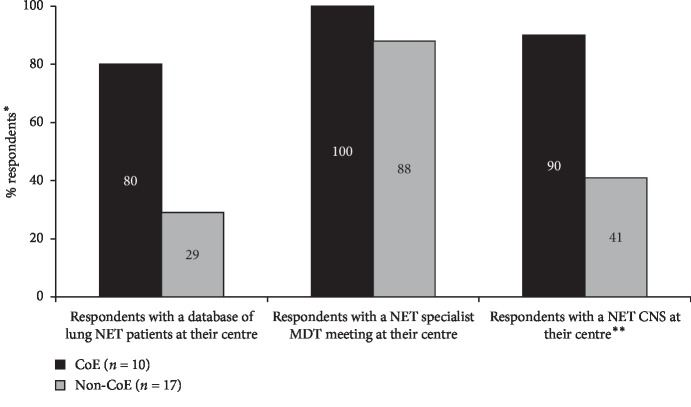
Comparison of reported patient management in CoE vs. non-CoE. ^*∗*^Bars represent the percentage of respondents reporting that they have a database of lung NET patients, NET specialist MDT meeting or NET CNS at their centre; the remainder reported ‘no' (i.e., database, NET specialist MDT meeting or NET CNS not available). ^*∗∗*^Respondents who reported that they have lung cancer CNS (but not a NET CNS) are not included. Abbreviations: CoE, Centre of Excellence; CNS, clinical nurse specialist; MDT, multidisciplinary team; NET, neuroendocrine tumour.

The disease management facilities reported to be available at CoE and non-CoE centres are shown in Figure 4. Overall, 93% (25/27) of the respondents reported having a NET-specialist MDT meeting for the management of patients with TC/AC (100% [10/10] of respondents from CoE and 88% [15/17] of respondents from non-CoE). Availability of a NET CNS was reported by 59% (16/27) of respondents overall (90% [9/10] of CoE and 41% [7/17] of non-CoE respondents), and availability of a lung NET patient database was reported by 48% overall (80% [8/10] of CoE and 29% [5/17] of non-CoE respondents).

## 4. Discussion

The results of this UK-wide survey of clinicians directly involved in patient care provide valuable insights into the diagnosis pathways and current management of patients with TC and AC in routine clinical practice in UK. Overall, the survey results demonstrate a degree of variability between centres in the diagnostic pathways and management of patients with TC and AC.

Although European (ENETS) best practice recommendations have been available since 2015 [8], before this survey was conducted, the variability demonstrated in our results suggests that these were not fully implemented across UK centres at the time of the survey. Furthermore, there has been a lack of consensus in recent years between different guidelines (e.g., ENETS [8] vs. National Comprehensive Cancer Network [NCCN] [18] and American Joint Committee on Cancer [AJCC] [19]) on the use of appropriate diagnostic assessment tools for TC and AC. In line with this, the results from this survey highlight variability among UK clinicians in the tests and investigations utilised for diagnosing TC and AC. Some consistencies were observed and in particular, the high use by respondents of the WHO classification, TNM staging, and chromogranin A for diagnosis is reassuring. A majority of respondents from CoE and non-CoE indicated that 5-hydroxyindoleacetic acid (5-HIAA) testing is routinely performed. Despite being considerably less common in pulmonary carcinoid tumours compared to GEP-NETs, 5-HIAA hypersecretion can occur and perhaps supports the practice of performing the test, given the ease of using a blood test and relative cost effectiveness. In the WHO classification, Ki67 scoring is not recommended for distinguishing between TC and AC but, nevertheless, appears to be widely used by clinicians in UK for diagnosis and to inform subsequent treatment decisions [25]. Recently, however, a UK-based study has shown that Ki67 levels, along with chromogranin A levels, age, and TNM stage, are significantly associated with patient survival [26]. In addition, in terms of follow-up, ENETS guidelines recommend a long-term follow-up, especially for patients with AC [8]. Our results have shown that whilst there was some evidence of more intensive follow-up of patients with AC (compared with TC), considerable variation in practice was evident, suggesting that long-term follow-up of patients is not routinely implemented. This is consistent with the results of two other published studies, which have also observed inconsistencies in follow-up surveillance in real-world practice, although both were conducted in single institutions (one in Ireland [27] and one in Italy [28]) before publication of the ENETS guidelines. Another study has also highlighted a similar lack of consensus for treatment options for pulmonary NETs across UK NHS centres [29]. Our results also suggest that CT scanning (the guideline-recommended imaging modality [8]) may not be utilised consistently in patient follow-up at all stages of the disease. In addition, an increase in the reported use of adjuvant treatment in later stages of the disease, as was shown in our results despite the lack of consensus in the available guidelines [8, 18, 30], underlines a need for more clarity on adjuvant therapies for these patients. Furthermore, the high proportion of respondents who believe that patients may be lost to follow-up in the current pathway is concerning, given the potential for late recurrence, and highlights the importance of an evidence-based approach to patient follow-up, with clear lines of responsibility.

The increasing variety of diagnostic and therapeutic approaches for patients with TC and AC [31], along with challenges in achieving accurate diagnosis and long-term follow-up of patients, has led to recommendations for a multidisciplinary approach from the care teams, involving collaboration between medical staff with a set of distinct expertise, to ensure each individual patient receives an optimal disease management plan [8]. It has been suggested that multidisciplinary care in specialised centres such as ENETS CoE [23], or the North American equivalent (multidisciplinary reference centres) [32], could improve clinical outcomes for patients with TC/AC. The European Reference Network (ERN) for rare tumours initiative will enhance the capability of sharing best practice across the continent [33].

We assessed the reported management pathways of patients in ENETS accredited CoE compared with nonaccredited UK centres. Although CoE were created for, and still work mainly on, GEP NETs, patients with pulmonary NETs will utilise the services set up by the CoE and in addition, ENETS has now extended its sphere of interest to include pulmonary NETs, for which it has published management guidance [8, 34]. Our results have shown that whilst all CoE and most non-CoE employ multidisciplinary teams for treating patients with AC/TC tumours, less than half (41%) of the non-CoE reported having a NET CNS on-site. Greater access to NET CNS and NET-specialist MDT meetings in CoE may result in improved supportive care for patients [35, 36]. Furthermore, though based on small numbers, our results suggest that CoE are more likely to use a wider range of diagnostic approaches compared to non-CoE, including greater use of ENETS-recommended tests and assessments such as gallium PET [8]. This could be due to having access to a wider range of diagnostic approaches in CoE compared to non-CoE, or perhaps a difference in the preferential use of particular diagnostic tools over the others across different centres. It is also acknowledged that some of the apparent differences between CoE and non-CoE could be interrelated; for example, the greater use of bone scans in CoE could be related to the wider use of gallium PET in these centres, which may contribute to increased identification of bone disease. Moreover, the finding that 80% of CoE, compared with only 29% of non-CoE, had a database of lung NET patients is important and suggests that CoE may also be better placed to support long-term patient follow-up. The apparent differences between CoE and non-CoE reported here likely reflect the rigorous auditing required for ENETS accreditation. Nevertheless, our results are broadly supportive of the CoE approach and suggest that these centres have an important role to play in optimising future patient management. Further studies are required to determine whether the apparent differences between CoE and non-CoE translate into improved patient outcomes and patient experience.

The variability seen in the results from this survey most likely reflect the current lack of consensus on the optimal diagnostic and treatment pathways for patients with TC and AC and highlight opportunities to optimise future patient management. The authors (WM and DT) are members of the UKI NETS team that has prepared a new clinical practice algorithm for the management of TC and AC, for which the current survey has proved informative. Whilst being broadly reflective of ENETS guidance, the algorithm is expected to provide comprehensive step-by-step guidance on the treatment choices available at different stages of the disease, including distinct guidelines for stage IV patients, and more detailed guidance respecting diagnosis, treatment options, and follow-up pathways for TC and AC. These guidelines will provide a practical tool for delivering recommended evidence-based practice for TC and AC and their implementation across all centres (CoE and non-CoE) alongside the existing ENETS guidelines may also help to reduce variability and standardise management of TC and AC in UK patients.

## 5. Strengths and Limitations

The survey has a number of strengths, including the wide geographical coverage of NET-specialist centres. In particular, the survey included respondents from all of the centres that had received ENET CoE accreditation at the time the survey was conducted. In addition, healthcare professionals were involved in designing the content of this survey to ensure all relevant areas were covered. Furthermore, the analysis was undertaken independently of the survey sponsor.

There are, however, some limitations in this survey. First, given the retrospective design and a lack of formal audit tools, the survey results may be subjected to individual bias. Second, as less than a third of the clinicians who were initially invited took part, results from the centres represented in the results may not reflect other UK centres. Furthermore, although the aim was to capture centre-level practice, the results may not fully reflect the opinions or practices of other clinicians within the participating centres as only one clinician per centre was interviewed. Third, the survey was not designed to investigate clinical outcomes (including survival) and the perspectives of patients with TC and AC. Although the interviewers were trained and the first interview was conducted jointly with two researchers, we cannot exclude the fact that the involvement of multiple interviewers could have led to differences in interpretation or variability in how the responses were collected. There were also a number of open-ended questions, which led to differences in the format and level of detail provided by the respondents. Most notably, information relating to follow-up practices was elicited using an open-ended question in which the respondents were asked how patients with resected TC/AC are followed up after completion of their primary treatment; in this instance, where information relating to the frequency, duration, or nature of patient follow-up was missing, it was not always apparent if this was because the information was not reported during the interview or if the absence of data was because the site did not perform this follow-up as standard.

## 6. Conclusions

The findings from this survey highlight the ongoing unmet challenges in the diagnosis and treatment of patients with TC and AC. The lack of consensus among UK clinicians on the use of diagnostic tools and treatment pathways, as well as considerable variation in the frequency and duration of patients' follow-up, calls for greater efforts to optimise the treatment of patients with TC and AC at specialised centres. In this regard, CoE may be able to offer an improved service to patients, as suggested by this survey. Studies comparing patient clinical outcomes and experiences between CoE and non-CoE and assessing the implementation of the new UKI NETS algorithm (the development of which was informed by the current survey) alongside ENETS guidelines across all specialist centres would help to assess whether CoE accreditation translates into measurable benefits for patients.

## Figures and Tables

**Table 1:** Proportion of respondents who would consider offering each type of adjuvant treatment to patients with TC or AC following surgical resection.

PC type/stage	Treatments^*∗*^ for patients with typical carcinoid tumours, no. (%) of respondents (*n* = 27)					Treatments^*∗*^ for patients with atypical carcinoid tumours, no. (%) of respondents (*n* = 27)				
	SSA	CT	RT	Treatment may be considered^*∗∗*^	Any type of treatment^#^	SSA	CT	RT	Treatment may be considered^*∗∗*^	Any type of treatment^#^
N0 M0	0 (0%)	0 (0%)	0 (0%)	1 (4%)	1 (4%)	0 (0%)	0 (0%)	0 (0%)	1 (4%)	1 (4%)
N1 M0	1 (4%)	0 (0%)	1 (4%)	3 (11%)	4 (15%)	2 (7%)	6 (22%)	2 (7%)	3 (11%)	9 (33%)
N2-3 M0	3 (11%)	2 (7%)	3 (11%)	3 (11%)	8 (30%)	2 (7%)	9 (33%)	2 (7%)	3 (11%)	13 (48%)
R1 resected	4 (15%)	2 (7%)	7 (26%)	2 (7%)	11 (41%)	3 (11%)	5 (19%)	7 (26%)	1 (4%)	13 (48%)

^*∗*^Treatment types not mutually exclusive. ^*∗∗*^Respondents who stated that the patient's suitability for adjuvant treatment may be discussed with the multidisciplinary team (or other specialist), but did not specify a particular type of treatment. ^#^Proportion of respondents who reported that they would consider any form of adjuvant treatment (including those who stated that the patient's suitability for treatment may be discussed with the multidisciplinary team [or other specialist]). CT: chemotherapy; PC: pulmonary carcinoid; RT: radiotherapy; and SSA: somatostatin analogue.

**Table 2:** Reported first-, second-, and third-line treatments for patients with advanced disease.

Treatments used (not mutually exclusive)	No. (%) of respondents (*n* = 27)		
	First-line	Second-line	Third-line
SSA	17 (63%)	5 (19%)	2 (7%)
CT	3 (11%)	9 (33%)	11 (41%)
Surgery	6 (22%)	—	1 (4%)
mTOR inhibitor/targeted therapy	1 (4%)	7 (26%)	3 (11%)
RT	—	2 (7%)	1 (4%)
PRRT	—	3 (11%)	5 (19%)
Interferon	—	—	1 (4%)
Clinical trial	—	1 (4%)	1 (4%)
Not known/not applicable^*∗*^	3 (11%)	2 (7%)	5 (19%)

^*∗*^Respondents who reported that they refer patients to another centre or specialist for treatment. CT: chemotherapy; mTOR: mammalian target of rapamycin; PRRT: peptide receptor radionuclide therapy; RT: radiotherapy; and SSA: somatostatin analogue.

**Table 3:** Reported follow-up frequency and total duration of follow-up in patients with TC and AC after completion of initial treatment.

PC type/stage	Initial follow-up frequency (range between respondents)		Total follow-up duration (range between respondents)	
	TC	AC	TC	AC
N0 M0	3–12 months	3–12 months	2–10 years	2–10 years
N1 M0	3–12 months	3–12 months	2–10 years	2–10 years
N2-3 M0	3–12 months	3–12 months	3–10 years	2 years–indefinite
R1 resected	3–12 months	3–12 months	3–10 years	2 years–indefinite
Stage IV	2–12 months	2–12 months	3 years–indefinite	3 years–indefinite

AC: atypical carcinoid; TC: typical carcinoid.

## Data Availability

The data used to support the findings of this study are included within the article.
